# Calibration of a Structured Light Imaging System in Two-Layer Flat Refractive Geometry for Underwater Imaging †

**DOI:** 10.3390/s23125444

**Published:** 2023-06-08

**Authors:** Domagoj Zoraja, Tomislav Petković, Josep Forest, Tomislav Pribanić

**Affiliations:** 1 University of Zagreb Faculty of Electrical Engineering and Computing, Unska 3, HR-10000 Zagreb, Croatia; tomislav.petkovic.jr@fer.hr (T.P.); tomislav.pribanic@fer.hr (T.P.); 2 Computer Vision and Robotics Research Institute (VICOROB), University of Girona, 17003 Girona, Catalunya, Spain; forest@eia.udg.edu

**Keywords:** calibration, structured light, underwater imaging, flat refractive geometry

## Abstract

The development of a robust 3D imaging system for underwater applications is a crucial process in underwater imaging where the physical properties of the underwater environment make the implementation of such systems challenging. Calibration is an essential step in the application of such imaging systems and is performed to acquire the parameters of the image formation model and to enable 3D reconstruction. We present a novel calibration method for an underwater 3D imaging system comprising a pair of cameras, of a projector, and of a single glass interface that is shared between cameras and projector(s). The image formation model is based on the axial camera model. The proposed calibration uses a numerical optimization of a 3D cost function to determine all system parameters, thus avoiding the minimization of re-projection errors which require numerically solving a 12th order polynomial equation multiple times for each observed point. We also propose a novel stable approach to estimate the axis of the axial camera model. The proposed calibration was experimentally evaluated on four different glass interfaces, wherein several quantitative results were reported, including the re-projection error. The achieved mean angular error of the system’s axis was under $6^{\circ }$, and the mean absolute errors for the reconstruction of a flat surface were 1.38 mm for normal glass interfaces and 2.82 mm for the laminated glass interface, which is more than sufficient for application.

## 1. Introduction

The applications of underwater 3D imaging systems span over various fields of research and have diverse applications, e.g., mapping of the underwater sites of archaeological value [1], monitoring of underwater wildlife in marine biology [2], and underwater inspection [3], to name a few. Recent developments also place such a system onto ROVs (remotely operated vehicles), UAVs (underwater autonomous vehicles), or similar underwater systems [4,5] thus making them perfect for underwater applications where human life might be endangered.

There are many challenges in the design and usage of an underwater 3D imaging system [6]. The difficulty of achieving the desired accuracy [7,8] of the imaging system is caused by image distortions due to the physical properties of light propagation in an underwater environment. To limit these, an underwater enclosure or a watertight housing must be designed accurately for each element of the imaging system, which include a camera and a projector. In the underwater environment, such enclosures introduce refractions and reflections of light-rays on both the air-to-glass interface and the glass-to-water interface, while water itself introduces attenuation and dispersion of the transmitted and reflected rays. Together, these effects present significant challenges in the calibration of a 3D imaging system. To overcome these challenges, cameras and projectors are usually first calibrated individually to acquire the corresponding intrinsic parameters, followed by a calibration of the whole 3D imaging system to acquire and to refine all parameters of the image formation model [9].

Sensors of a 3D imaging system may be classified as passive or active. The key difference is that active 3D imaging introduces an artificial illumination in the observed scene. A typical example of passive 3D imaging is stereo vision [10], and an example of active 3D imaging is structured light or SL [11,12]. In an SL imaging system, a projector projects a code onto a surface of an observed object, which is then observed by one or more cameras to reconstruct the 3D shape. Therefore, an underwater 3D imaging system comprising one or more cameras and one or more projectors is an active SL 3D imaging device for which there are many reported uses in underwater imaging [1,3,6,13,14,15]. Next, underwater SL systems may be classified depending on the shape of the air-to-water interface, usually a protective glass, which may be flat or curved [9,16]. Although curved interfaces offer some advantages, including the compensation of unwanted refractions, they are difficult to design and to manufacture, thus making flat interfaces a more convenient and more flexible choice. Finally, when a camera or a projector is coupled with a flat refractive interface, the ubiquitous pinhole camera model [10,17,18] is no longer applicable and must be extended. One possible extension of the pinhole model for flat refractive interfaces is the axial camera model [19,20], which can account for the spatial spreading of the focal point of the pinhole camera model.

In this paper, we present a novel method for calibrating a structured light 3D imaging system, which comprises two cameras and of one projector and which uses a shared flat refractive interface (a single shared glass). The proposed method is a continuation of our previous work [9] on projector calibration for underwater imaging, which we here extend to the calibration of the whole SL imaging system. It is also based on the previous work by Agrawal et al. [19], which introduced flat refractive imaging geometry for a single camera, which we extended to a system of two cameras and a projector. The proposed calibration method uses a planar calibration board with white circles arranged in a hexagonal pattern as a calibration object [21]. Once the calibration object is imaged in several positions, the obtained data is combined with the in-the-air calibration data to obtain the axis of the axial camera first, which is then followed by a numerical optimization procedure using a 3D cost function to determine the final imaging parameters of the SL system. The proposed calibration method was thoroughly evaluated in a laboratory environment for four different glass interfaces. To summarize, compared to the previous works and to the state-of-the-art approaches, our contributions are the following:

1. The coplanarity constraint proposed by Agrawal et al. [19] and used in our previous work [9] was extended to the case of multiple cameras and projectors that share a single flat glass interface and was then applied to the estimation of the axial camera’s axis. The proposed axis estimation was more stable than the method of [19] as demonstrated by the performed experiments.
2. The proposed optimization of the 3D cost function from our previous work [9] was extended to the whole SL imaging system, and we also introduced boundaries on allowed system parameters.
3. Calibration using the extended coplanarity constraint and the proposed 3D cost function were thoroughly evaluated on four different glass types, and the obtained results verify that the proposed method can cope with differing glass thicknesses.

Note that, to the best of our knowledge, the proposed method is the first method for calibration of a SL underwater imaging system that uses a single glass interface.

The rest of this paper is structured as follows: Section 2 describes state-of-the-art and related works. In Section 3, we present the proposed calibration method. Experimental results are presented in Section 4 and are discussed in Section 5. We conclude with Section 6.

## 2. Related Work

In this section, we present prior works considering the in-the-air geometric calibration of multiple cameras and projectors based on the work by Petković et al. [21], which follows with an individual camera (or projector) calibration for the underwater imaging, where a camera is represented using an axial parametric model with an additional calibration of the refractive parameters as proposed by Agrawal et al. [19]. This approach has been extended to projector calibration by Zoraja et al. [9]. In addition, we present prior works where a camera was modeled purely by the pinhole model [17], without any additional imaging parameters to model refractions, and by the Pinax model [22], which allows a fast refraction correction of the flat pane housing by utilizing a pre-computed lookup table.

The geometric calibration of multiple cameras and projectors for in-the-air imaging is an essential step in the everyday use of an SL imaging system [11]. A typical calibration procedure utilizes a planar calibration object that comprises bright circles on a dark background arranged in a regular lattice and follows a flexible method proposed by Zhang and Huang [23] for projector calibration. Such a calibration board makes the projected code easier to decode w.r.t. the standard checkerboard pattern. Petković et al. [21] summarized the whole procedure and also proposes to use a hexagonal calibration lattice, as it increases the number of calibration points, and a multiple phase shift (MPS) structured light code, as it inherently provides subpixel precise mapping w.r.t. the gray code, which requires interpolation for such a level of precision.

**Figure 1:**
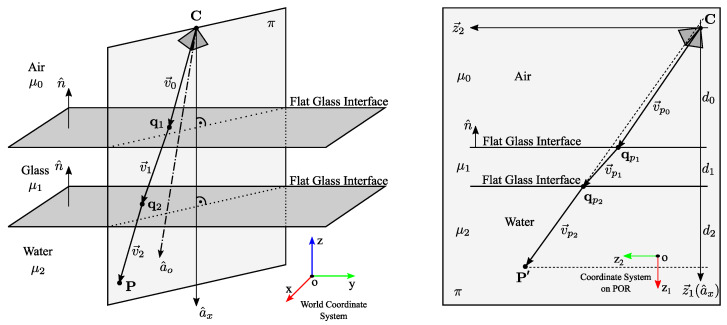
A two-layer flat refractive geometry and a plane of refraction π. **Left**: 3D view. **Right**: plane of refraction.

A theory of multi-layer flat refractive geometry proposed by Agrawal et al. [19] provides the basis for underwater camera calibration. Their calibration procedure is based on a pinhole camera model, which is extendend to an axial model with additional parameters to model refractions on flat layers. The camera is firstly calibrated in air using Zhang’s [24] calibration procedure, followed by the underwater imaging of a planar calibration object to determine additional refractive parameters. Agrawal et al. introduced a multi-layer flat refractive geometry and a plane of refraction (POR), which have been used in our calibration method as well. They model the imaging system as an axial camera [20], since all refracted camera rays when extended to infinity intersect the axis at some point. Here, we stress the fact that the axis of the whole system is not the optical axis of a single camera, although, in a special case, they may coincide. The coplanarity constraint for a single camera yields a linear system, which is solved using singular value decomposition (SVD) to yield an essential matrix from which the axis of the axial system is determined. The pose parameters R and t are also computed from the essential matrix, and the layers’ thicknesses $d_{i}$ are computed using the flat refraction constraint (FRC), which states that the segment between the 3D point P and the point on the last refractive layer ${\vec{q}}_{2}$ should be parallel to the final refracted ray ${\vec{v}}_{2}$ (see Figure 1). The refinement of all calibration and pose parameters is achieved by minimizing the re-projection error, which requires solving the analytical forward projection (AFP) equation. The AFP describes a method for the analytical computation of the projection of a known 3D point and is computationally expensive, which has prompted further research into possible simplifications, as performed by Kawahara et al. [25]. Our proposed calibration method sidesteps this issue by using a 3D cost function instead of the AFP.

A projector calibration in a two-layer flat refractive geometry presented by Zoraja et al. [9] provides a projector calibration procedure where the projector is modeled as an inverse camera. The projector calibration proposed by Zoraja et al. [9] utilizes the geometric camera calibration procedure proposed by Petković et al. [21] and an additional calibration of the refractive parameters as proposed by Agrawal et al. [19]. Zoraja et al. [9] also discussed the noted instability in the estimation of the axis of an axial system as proposed by Agrawal et al. [19]; this instability has been addressed in our calibration method.

An underwater 3D reconstruction using structured light was proposed by Bruno et al. [13], which provided experimental results for a highly turbid environment with a heavy presence of scattering and absorption. Their imaging system was experimentally assessed in different turbidity conditions. For the underwater calibration, Bruno et al. [13] used Bouguet’s Camera Toolbox [26] and adopted the ubiquitous in-the-air calibration model; thus, they did not consider optical properties of different mediums, including air and water. Such a calibration procedure utilizing only the pinhole camera model with standard distortions, without any additional calibration of the refractive parameters, is simple and convenient to use, but it has a larger error than a proper model, which includes refractions [19]. Here, we note that the necessary requirements [22,27] for the pinhole model to be applicable are that the glass interface should be: (1) perpendicular to the camera’s optical axis, (2) be as close to the lens as possible, and (3) be as thin as possible.

Finally, the Pinax model for accurate and efficient refraction correction, presented by Łuczyński et al. [22], allows for a pre-computation of a lookup-table for very fast refraction correction of the flat-pane with high accuracy. The model takes the refraction indices of water into account, especially with respect to salinity, and it is, therefore, sufficient to calibrate the underwater camera only once in-the-air. They require that the optical axis of the camera be perpendicular to the glass surface and that the distance $d_{0}$ (see Figure 1) between the glass and the center of projection be minimal; the posed requirements were not considered in our implementation i.e., the direction of camera’s (projector’s) optical axis and the distance $d_{0}$ was considered to be arbitrary.

## 3. Calibration Method

The proposed calibration method is applicable to a structured light imaging system comprising an arbitrary number of cameras and projectors that all share a single flat interface that separates them from the water. We first provide a description of the imaging geometry used in Section 3.1, which is followed by a description of the coplanarity constraint for an individual camera (or projector) in Section 3.2, which is then extended to an arbitrary number of cameras and projectors in Section 3.3). The in-the-air calibration of individual cameras and projectors and of a whole SL system are described in Section 3.4. Finally, the proposed numerical optimization using a 3D cost function is described in Section 3.5, and the final calibration procedure of a SL system is described in Section 3.6.

### 3.1. Imaging Geometry

The building blocks of an SL imaging system are cameras and projectors, and, for underwater imaging, we also require a protective flat glass. W.l.o.g., we limit the discussion to a system comprising two cameras and of one projector. The protective interface must be flat and must be shared between all cameras and projectors in the imaging system. Then, the imaging geometry of the SL imaging system is based on the multi-layer flat refractive geometry introduced by Agrawal et al. [19]. For a submerged imaging system enclosed in a watertight housing using a single protective glass, there are two interfaces between mediums with different optical properties: an air-to-glass interface and a glass-to-water interface. This two-layer flat refractive geometry is presented in Figure 1. On the left, ${\vec{v}}_{i}$ represents the light path of a camera/projector ray in 3D (for a two-layer system i=0,1,2): ${\vec{v}}_{0}$ is the initial camera ray, ${\vec{v}}_{1}$ and ${\vec{v}}_{2}$ are refracted rays, and $q_{i+1}$ are points of refraction. A point *P* is the point in the 3D space, and *C* is the extrinsic center of a pinhole camera/projector model. The axis of the axial camera model ${\hat{a}}_{x}$ points in the opposite direction of the refractive interface’s normal $\hat{n}$. Note that ${\hat{a}}_{o}$ denotes the optical axis of the camera, which is different than the axis ${\hat{a}}_{x}$.

A plane of refraction π introduced by [19] is the most important concept in the multi-layer flat refractive geometry, as it contains the entire light path of a camera ray for each pixel (see Figure 1, right). When considering the properties of the light propagation, both the incident ray and the surface normal lie on the unique plane π, and when considering Snell’s law, the refracted ray must also lie on π; hence, by induction, the entire light path and the point *P* are also on π [19].

A 2D coordinate system on the POR is defined by a vector ${\vec{z}}_{1}$, which points in the direction of the axis ${\hat{a}}_{x}$ of the axial system, and a vector ${\vec{z}}_{2}$, which is defined for an unique camera ray ${\vec{v}}_{0}$ as ${\vec{z}}_{2}={\vec{z}}_{1}\times \left({\vec{z}}_{1}\times {\vec{v}}_{0}\right)$. A typical POR π for an unique pixel is shown in Figure 1: on the right, it contains the axis ${\hat{a}}_{x}$, the light path ${\vec{v}}_{p_{i}}$ of a camera/projector ray in 2D, points of refraction $q_{p_{i+1}}$, and the point $P^{\prime }$ denoting the point *P* in the coordinate system on the POR. Note that ${\vec{v}}_{p_{i}}$ (see Figure 1, right) denotes the camera/projector rays ${\vec{v}}_{i}$ (see Figure 1, left) in the coordinate system on the POR. Vector $d_{i}$ represents the layers’ thickness; $d_{0}$ is the distance between camera’s (projector’s) extrinsic center to the boundary of the first refractive interface; $d_{1}$ is the thickness of the second interface (glass interface); and $d_{2}$ is the distance between the boundary of the second refractive interface and the point $P^{\prime }$. Note that, for each pixel, there exists a unique POR and that all PORs for all corresponding pixels comprise a pencil of planes through the straight line, which is the axis of the axial camera ${\hat{a}}_{x}$ [9,20].

### 3.2. The Coplanarity Constraint for a Camera/Projector Using a Single Interface

**Figure 2:**
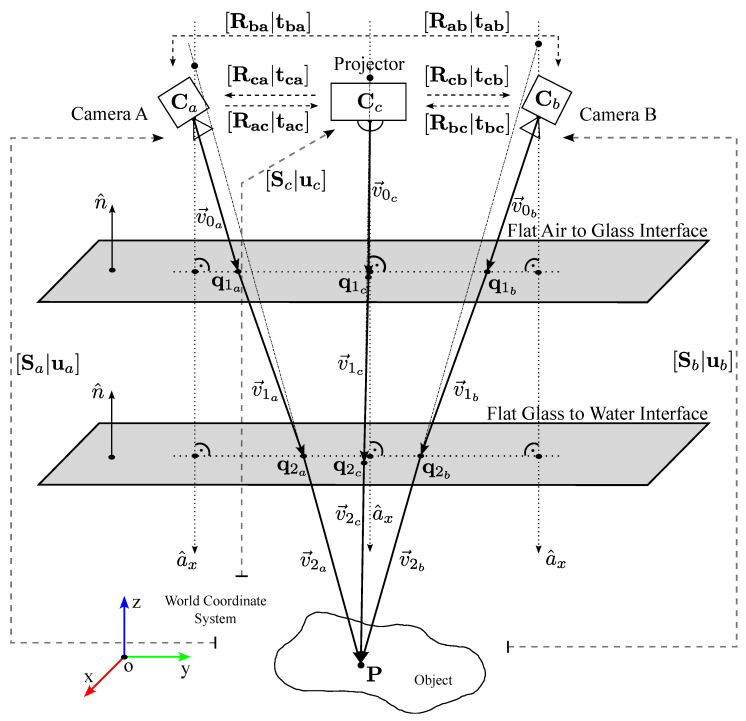
Relative poses of cameras and a projector w.r.t. the world coordinate system.

A constraint that binds the point *P* and the corresponding initial camera ray ${\vec{v}}_{0}$ to the same plane (POR) is called the *coplanarity* constraint [19], and it may be expressed in a matrix form as

(1) $${\left({\left[a\right]}_{\times }v_{0}\right)}^{T}\left(Sp+u\right)=0,$$

where ${\left[a\right]}_{\times }$ is a skew-symmetric matrix representing ${\hat{a}}_{x}$ in the camera coordinate system, $v_{0}$ is a 3×1 vector representing the initial camera ray ${\vec{v}}_{0}$ in the camera coordinate system, and p is a 3×1 vector representation of the illuminated point *P* in the calibration frame. As Equation (1) is expressed in the camera coordinate system, the parameters S (rotation) and u (translation) represent the relative pose of the world coordinate system to the camera coordinate system (see Figure 2). The coplanarity constraint that binds a relative pose of the world coordinate system to the camera coordinate system may be re-written as

(2) $$\begin{array}{cc} v_{0}^{T}{\left[a\right]}_{\times }Sp+v_{0}^{T}{\left[a\right]}_{\times }u & =0. \end{array}$$

The product of a skew-symmetric matrix ${\left[a\right]}_{\times }$ and a rotation matrix S is a 3×3 a matrix E, which has the properties of an essential matrix [10]; note that this is not an essential matrix of a stereo vision system. The product of a skew-symmetric matrix ${\left[a\right]}_{\times }$ and a translation vector u is a 3×1 vector h representing a constraint on the translation vector u. Considering ${\left[a\right]}_{\times }S=E$ and ${\left[a\right]}_{\times }u=h$, Equation (2) may be expressed as

(3) $$v_{0}^{T}Ep+v_{0}^{T}h=0,$$

and, by forming a Kronecker’s product (denoted as ⊗) between points p(i) in 3D space and their corresponding initial camera rays $v_{0}\left(i\right)$, we may express Equation (3) as a linear system by stacking equations for their points’ correspondences

(4) $$\underset{Q}{\underset{︸}{\left[\begin{array}{cc} p{\left(1\right)}^{T}⊗v_{0}{\left(1\right)}^{T} & v_{0}{\left(1\right)}^{T}\\ p{\left(2\right)}^{T}⊗v_{0}{\left(2\right)}^{T} & v_{0}{\left(2\right)}^{T}\\ \vdots & \vdots \\ p{\left(N\right)}^{T}⊗v_{0}{\left(N\right)}^{T} & v_{0}{\left(N\right)}^{T} \end{array}\right]}}\left[\begin{array}{c} E(:)\\ h \end{array}\right]\;=0.$$

We denote a N×9 matrix of the linear system with Q. *N* is the number of points’ correspondences. Vector E(:) is a column vector formed by stacking columns of the essential matrix E.

The solution of the linear system, given by Equation (4), is acquired by performing a singular value decomposition (SVD) of the matrix Q. A planar calibration object is commonly represented as the xy plane with the *z* coordinate set to zero [24], so $p={\left[x_{i},y_{i},0\right]}^{T}$ and columns 7, 8, and 9 all reduce to zero. Therefore, we cannot directly estimate the full matrix E; we may only estimate the first two columns of E. The last column of E is estimated using Demazure’s [28] constraints (also see Nister [29]). The axis a of the axial system, a 3×1 vector, may be extracted from the acquired E matrix by performing an SVD. Note that $h^{T}a=0$; hence, the full translation cannot be extracted, as the component of u in the direction of the axis vanishes in h. By using the acquired solution for h, we may only estimate a part of translation orthogonal to the axis $u_{\mathrm{orth}}$ as a cross product of h and a. Note that the full translation may be estimated from a flat refraction constraint (FRC) defined on the POR for each point correspondence; see Agrawal et al. [19]. Alternatively, a central approximation may be used to obtain an approximate estimate. In a central approximation, 3D points in the world frame are mapped to 2D points in the image frame by a central projection model in which a ray defined by a 3D point in space and a fixed point in space, the center of projection, intersects a specific plane in space that is chosen as the image plane (see Hartley and Zisserman [10]). In our calibration procedure, the rotation and translation estimates were computed using central approximation, which is simpler than solving the FRC and which yields appropriate initial points for the numerical optimization.

The coplanarity constraint may be derived separately for each pose of the camera/projector w.r.t. the calibration object. In the case of multiple cameras/projectors, we set the coordinate system of the camera a as the referent one; hence, the coplanarity constraint may be re-written w.r.t. to this referent frame as

(5) $$\begin{array}{cc} v_{0_{a}}^{T}\left[{\left[a_{1}\right]}_{\times }S_{a}p_{a}+{\left[a_{1}\right]}_{\times }u_{a}\right] & =0, \end{array}$$

(6) $$\begin{array}{cc} v_{0_{b}}^{T}\left[{\left[R_{\mathrm{ab}}a_{1}\right]}_{\times }\left(R_{\mathrm{ab}}S_{a}p_{b}+R_{\mathrm{ab}}u_{a}+t_{\mathrm{ab}}\right)\right] & =0, \end{array}$$

(7) $$\begin{array}{cc} v_{0_{c}}^{T}\left[{\left[R_{\mathrm{ac}}a_{1}\right]}_{\times }\left(R_{\mathrm{ac}}S_{a}p_{c}+R_{\mathrm{ac}}u_{a}+t_{\mathrm{ac}}\right)\right] & =0, \end{array}$$

where Equation (5) represents the coplanarity constraint of the camera a, Equation (6) is the coplanarity constraint of the camera b in the frame a, and Equation (7) denotes the coplanarity constraint of the projector in the frame a (see Figure 2). Each constraint yields a certain pose of the camera (projector) w.r.t. the calibration object: the pose $\left[S_{a}|u_{a}\right]$ for the correspondence between the object’s frame and the frame a, the pose $\left[S_{b}|u_{b}\right]$ for the correspondence between the object’s frame and the frame b, and the pose $\left[S_{c}|u_{c}\right]$ for the correspondence between the object’s frame and the frame c.

The rotation matrix $R_{\mathrm{ab}}=R_{b}{R_{a}}^{T}$ is the rotation from the camera coordinate system a to the frame b, and $R_{\mathrm{ac}}=R_{c}{R_{a}}^{T}$ is the rotation from the camera coordinate system a to the projector’s frame (frame c). The corresponding translations are $t_{\mathrm{ab}}=-R_{b}C_{b}+R_{b}C_{a}$ and $t_{\mathrm{ac}}=-R_{c}C_{c}+R_{c}C_{a}$, where $C_{i}$ denotes the extrinsic center of the corresponding camera (projector). These rotations and translations are determined during the in-the-air calibration. The axis $a_{1}$ of an axial system is the system’s axis expressed in the referent frame (frame a). In Equations (6) and (7), the axes $a_{2}$ and $a_{3}$ are rotated into the referent frame, since $a_{2}=R_{\mathrm{ab}}a_{1}$ and $a_{3}=R_{\mathrm{ac}}a_{1}$.

The rotation matrices $S_{b}$ and $S_{c}$ w.r.t. the frame a are $S_{b}=R_{\mathrm{ab}}S_{a}$ and $S_{c}=R_{\mathrm{ac}}S_{a}$. The corresponding translations w.r.t. the frame a are $u_{b}=R_{\mathrm{ab}}u_{a}+t_{\mathrm{ab}}$ and $u_{c}=R_{\mathrm{ac}}u_{a}+t_{\mathrm{ac}}$. The $3\times N_{i}$ matrices of initial camera rays are denoted with $v_{0_{a}}$, $v_{0_{b}}$, and $v_{0_{c}}$, and they are expressed in the corresponding camera/projector frame (see Figure 2). The corresponding $p_{i}$ denotes $3\times N_{i}$ matrices of the object’s points in the world coordinate system (the object’s frame). Note that the coplanarity constraint is performed for each pose of the calibration object. For a complete derivation of the coplanarity constraint, see Appendix A.1.

### 3.3. Unified Coplanarity Constraint for a System Using a Single Interface

Following up on Section 3.2, we proposed to calibrate the system by using an unified coplanarity constraint. We formed the coplanarity constraint equations separately for each pose of the camera/projector w.r.t. the calibration board (see Equations (8)–(10)), and we combined them into a single linear equation for the whole system (see Equation (14)). Considering ${\left[a\right]}_{\times }R=E$ and ${\left[a\right]}_{\times }t=h$, Equations (5)–(7) may be simplified as

(8) $$\begin{array}{ccccc} v_{0_{a}}^{T}E_{1}p_{a} & + & v_{0_{a}}^{T}h_{1} & & =0 \end{array}$$

(9) $$\begin{array}{ccccc} v_{0_{b}}^{T}R_{\mathrm{ab}}E_{1}p_{b} & + & v_{0_{b}}^{T}R_{\mathrm{ab}}h_{1} & +v_{0_{b}}^{T}{\left[R_{\mathrm{ab}}a_{1}\right]}_{\times }t_{\mathrm{ab}} & =0 \end{array}$$

(10) $$\begin{array}{ccccc} v_{0_{c}}^{T}R_{\mathrm{ac}}E_{1}p_{c} & + & v_{0_{c}}^{T}R_{\mathrm{ac}}h_{1} & +v_{0_{c}}^{T}{\left[R_{\mathrm{ac}}a_{1}\right]}_{\times }t_{\mathrm{ac}} & =0, \end{array}$$

where the essential matrix $E_{1}$, the parameter $h_{1}$, and the axis $a_{1}$ denote the unknown parameters in the frame a (the referent frame). Furthermore, we may refactor the term $v_{0_{b}}^{T}{\left[R_{\mathrm{ab}}a_{1}\right]}_{\times }t_{\mathrm{ab}}$ in Equation (9) as ${\left[{R_{ab}}^{T}\left(t_{\mathrm{ab}}\times v_{0_{b}}\right)\right]}^{T}a_{1}$ and the term $v_{0_{c}}^{T}{\left[R_{\mathrm{ac}}a_{1}\right]}_{\times }t_{\mathrm{ac}}$ in Equation (10) as ${\left[{R_{ac}}^{T}\left(t_{\mathrm{ac}}\times v_{0_{c}}\right)\right]}^{T}a_{1}$, thus forming a linear system

(11) $$\begin{array}{ccccc} v_{0_{a}}^{T}E_{1}p_{a} & + & v_{0_{a}}^{T}h_{1} & & =0 \end{array}$$

(12) $$\begin{array}{ccccc} v_{0_{b}}^{T}R_{\mathrm{ab}}E_{1}p_{b} & + & v_{0_{b}}^{T}R_{\mathrm{ab}}h_{1} & +{\left[{R_{ab}}^{T}\left(t_{\mathrm{ab}}\times v_{0_{b}}\right)\right]}^{T}a_{1} & =0 \end{array}$$

(13) $$\begin{array}{ccccc} v_{0_{c}}^{T}R_{\mathrm{ac}}E_{1}p_{c} & + & v_{0_{c}}^{T}R_{\mathrm{ac}}h_{1} & +{\left[{R_{ac}}^{T}\left(t_{\mathrm{ac}}\times v_{0_{c}}\right)\right]}^{T}a_{1} & =0, \end{array}$$

which may be refactored further w.r.t. Equation (3), by using Kronecker’s product, and represented in the matrix form:

(14) $$\left[\begin{array}{ccc} p_{a}{\left(1\right)}^{T}⊗v_{0_{a}}{\left(1\right)}^{T} & v_{0_{a}}{\left(1\right)}^{T} & 0\\ p_{a}{\left(2\right)}^{T}⊗v_{0_{a}}{\left(2\right)}^{T} & v_{0_{a}}{\left(2\right)}^{T} & 0\\ \vdots & \vdots & \vdots \\ p_{b}{\left(1\right)}^{T}⊗\left(v_{0_{b}}{\left(1\right)}^{T}R_{\mathrm{ab}}\right) & v_{0_{b}}{\left(1\right)}^{T}R_{\mathrm{ab}} & {\left[{R_{ab}}^{T}\left(t_{\mathrm{ab}}\times v_{0_{b}}\left(1\right)\right)\right]}^{T}\\ p_{b}{\left(2\right)}^{T}⊗\left(v_{0_{b}}{\left(2\right)}^{T}R_{\mathrm{ab}}\right)\; & v_{0_{b}}{\left(2\right)}^{T}R_{\mathrm{ab}} & {\left[{R_{ab}}^{T}\left(t_{\mathrm{ab}}\times v_{0_{b}}\left(2\right)\right)\right]}^{T}\\ \vdots & \vdots & \vdots \\ p_{c}{\left(1\right)}^{T}⊗\left(v_{0_{c}}{\left(1\right)}^{T}R_{\mathrm{ac}}\right) & v_{0_{c}}{\left(1\right)}^{T}R_{\mathrm{ac}} & {\left[{R_{ac}}^{T}\left(t_{\mathrm{ac}}\times v_{0_{c}}\left(1\right)\right)\right]}^{T}\\ p_{c}{\left(2\right)}^{T}⊗\left(v_{0_{c}}{\left(2\right)}^{T}R_{\mathrm{ac}}\right)\; & v_{0_{c}}{\left(2\right)}^{T}R_{\mathrm{ac}} & {\left[{R_{ac}}^{T}\left(t_{\mathrm{ac}}\times v_{0_{c}}\left(2\right)\right)\right]}^{T}\\ \vdots & \vdots & \vdots \end{array}\right]\left[\begin{array}{c} E_{1}\left(:\right)\\ h_{1}\\ a_{1} \end{array}\right]=0$$

Equation  (14) is the unified coplanarity constraint for a system using a single interface w.r.t. the coordinate system of the camera a. Since we are using a planar calibration object, only the first two columns of the essential matrix $E_{1}$ may be estimated using SVD; the third column is estimated using the Demazure’s [28] constraints (also see Nister [29]). Note that the key feature in the proposed unified coplanarity constraint is the estimation of the system axis ${\hat{a}}_{x}$ (expressed in the referent frame as axis $a_{1}$) derived directly from the posed linear system (Equation 14). The estimated parameter $h_{1}$ is not used, since we compute the initial pose parameters directly from the central approximation. Note that the unified coplanarity constraint is performed for each pose of the calibration object, thereby implying that we acquire as many solutions as there are positions of the imaged calibration object. The system’s axis is then acquired as a circular mean of the estimated axes. For a full derivation of the unified coplanarity constraint, see Appendix A.2.

### 3.4. In-the-Air Calibration

In-the-air geometric calibration is required in order to determine the intrinsic parameters of camera/projector and the relative poses between cameras and projectors [21]. This geometric calibration is a necessary prerequisite for the proposed calibration of the underwater imaging system and is performed in three steps:

1. Create an image of a planar calibration board in many positions;
2. Extract the image coordinates of the calibration points;
3. Optimize all the parameters by minimizing the re-projection error.

Regarding projector calibration, the projector is not able to acquire images of the observed illuminated scene. The necessary data is instead acquired by cameras in the imaging system [21,23,30]. Since the projector is modeled as an inverse camera, the standard pinhole camera model with radial distortion is used. The state-of-the-art projector calibration algorithms follow the procedure proposed by Zhang and Huang [23] using a structured light scanning technique to obtain calibration data [11,12]. The projector’s row and column coordinates are embedded in the projected coded pattern, which yields direct correspondences between an uncalibrated camera and the projector [21].

**Figure 3:**
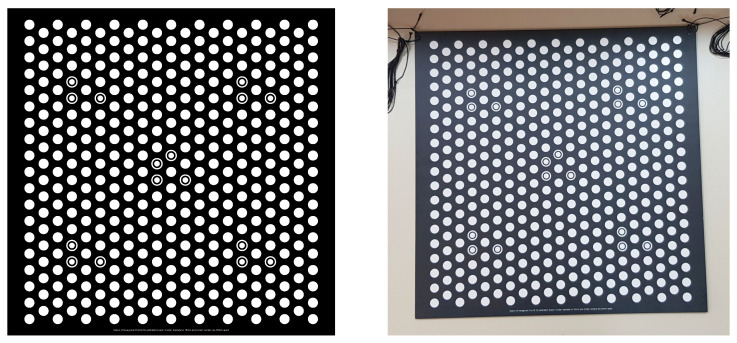
Planar calibration board (**left**: ideal calibration pattern; **right**: physical board used).

Considering the calibration object, we used a planar calibration board with a regular hexagonal lattice of bright circles on a dark background, which is shown in Figure 3. The projected SL code is easier to decode for white circles when compared to the standard checkerboard pattern [30]. The calibration board should be imaged in many different positions w.r.t. the camera/projector position [21]. For each camera and projector, two different positions of the calibration board constitute the minimum [24]. We proposed to use MPS-coded patterns, which enable simultaneous acquisition in a system comprising of an arbitrary number of projectors [31,32]. Another key point of the proposed approach is that, if there are multiple cameras observing the same part of the calibration board illuminated by a projector, then the number of extracted points for calibrating that particular projector is increased as measurements are repeated up to the number of cameras observing the illuminated board [21].

### 3.5. Numerical Optimization

As proposed in Section 3.3, we did not estimate the pose parameters [S|u] from the coplanarity constraint (1); instead, we utilized a central approximation algorithm to acquire the initial pose of the camera(s) and projector(s). To refine the acquired pose parameters, we designed a numerical optimization algorithm, which follows our previous implementation [9], which was extended to include parameter boundaries. Instead of minimizing the re-projection error, we minimized the 3D cost function of [9], which we describe here for completeness. This avoided the minimization of the re-projection error, which introduces high computational requirements of an analytical forward projection algorithm for which a 12th degree polynomial must be solved for each calibration point and for each iteration of the minimization [19,25].

The total error in 3D comprises three components: (1) coplanarity error; (2) frustum error; and (3) backprojection error. Posed errors may be used to form an objective function in a nonlinear least-squares minimization algorithm.

#### 3.5.1. Coplanarity Error

The coplanarity constraint (1) limits the position of a point $\vec{p}$ and its corresponding ray ${\vec{v}}_{0}$ to an unique plane (the POR, see Figure 1). The shortest vector ${\vec{r}}_{\mathrm{CPL}}$ connecting the point $\vec{p}$ to the plane is

(15) $${\vec{r}}_{\mathrm{CPL}}=\left(\delta -\hat{n}\cdot \vec{p}\right)\;\hat{n},$$

where a plane in the Hesse normal form is described by its normal $\hat{n}$ and its signed distance to the origin δ. It is implied that a posed constraint must be zero for all points and their corresponding rays; hence, the sum of squares of two-norms is

(16) $$\sum_{i}\left|\right|{\vec{r}}_{\mathrm{CPL},i}{\left|\right|}_{2}^{2}$$

for all ray-point correspondences $\left({\vec{v}}_{0,i},{\vec{p}}_{i}\right)$ must be minimized in order to find the local minimum.

#### 3.5.2. Frustum Error

**Figure 4:**
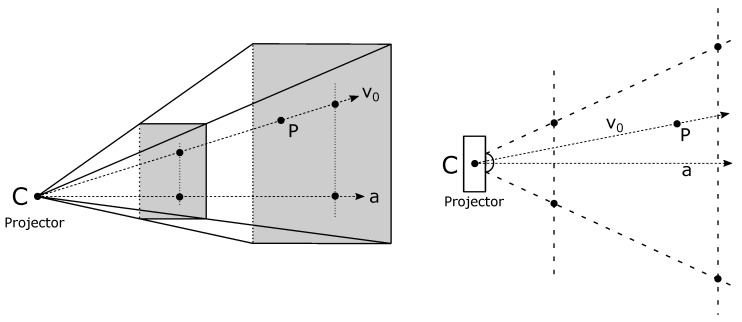
Projector’s clipping planes (**left**: 3D view; **right**: top 2D view).

Since all calibration points must be in a field of view (FoV), we proposed a frustum error [9] as the distance to the projector’s clipping planes (see Figure 4). Frustum is defined by six clipping planes: front, back, top, bottom, left, and right. We may apply a constraint by considering the distance to a clipping plane, with the purpose to guide the minimization by constraining the pose parameters. The equations of all six clipping planes are easily expressed in the Hesse normal form from the known in-the-air calibration. If the normals of all the clipping planes are oriented to point inside the frustum, then the vector ${\vec{r}}_{\mathrm{FRS}}$ connecting the point $\vec{p}$, which is outside of the frustum to the clipping plane, $\left({\hat{n}}_{j},\delta_{j}\right)$ is

(17) $${\vec{r}}_{\mathrm{FRS}}=\left\{\begin{array}{cc} \left(\delta_{j}-{\hat{n}}_{j}\cdot \vec{p}\right){\hat{n}}_{j}, & {\hat{n}}_{j}\cdot \vec{p}-\delta_{j}>0\\ 0, & \mathrm{else}. \end{array}\right.$$

Equation (17) implies that, if the point is on the correct side of the clipping plane (inside the frustum), then we set ${\vec{r}}_{\mathrm{FRS}}$ to zero. All calibration points that end up outside of or on the boundaries of the frustum are fed to the numerical optimization algorithm. The sum of squares of two-norms is

(18) $$\sum_{i}\left|\right|{\vec{r}}_{\mathrm{FRS},i}{\left|\right|}_{2}^{2},$$

which defines the objective function in a minimization scheme.

#### 3.5.3. Backprojection Error

**Figure 5:**
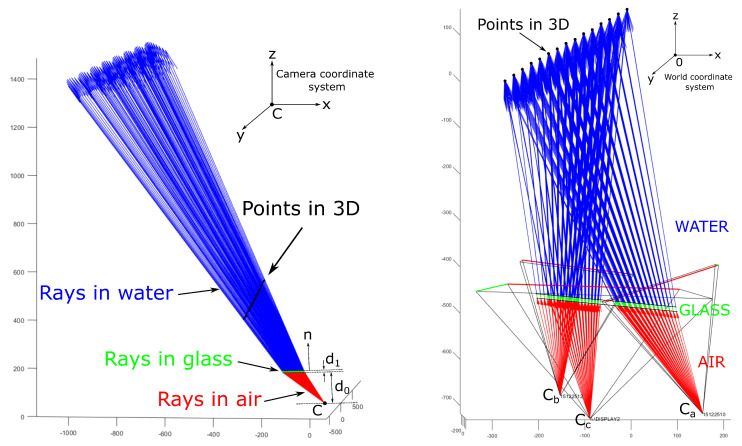
Initial and refracted camera rays. **Left**: for a single camera in the corresponding camera coordinate system. **Right**: for a system in the world coordinate system. Initial camera rays in the air are displayed in red, rays refracted in the glass are displayed in green, and final refracted rays in the water are displayed in blue.

From the known imaging geometry, we may analytically derive the equations of refracted rays in the water [19]. We are only interested in the part of the last ray in the water, which is inside the projector’s frustum. This ray segment is best modeled via its two endpoints, which are denoted as ${\vec{q}}_{2}$ and ${\vec{q}}_{3}$ (see Figure 1 and Figure 5):

(19) $${\vec{q}}_{3}={\vec{q}}_{2}+\ell {\hat{v}}_{2},$$

where *ℓ* represents the distance between ${\vec{q}}_{2}$ and ${\vec{q}}_{3}$, which is defined by the projector’s frustum. The point ${\vec{q}}_{2}$ denotes the point of refraction on the second refractive layer, and ${\vec{q}}_{3}$ is the point on the camera ray in the 3rd medium (water). The distance of a point ${\vec{p}}_{i}$ to the line segment connecting ${\vec{q}}_{2}$ to ${\vec{q}}_{3}$ is defined as the backprojection error. Expressing the line in a parametric form as ${\vec{q}}_{2}+t{\vec{q}}_{3}$ enables a simple computation of *t*, which defines the closest point as

(20) $$t=\frac{\left({\vec{p}}_{i}-{\vec{q}}_{2}\right)\cdot \left({\vec{q}}_{3}-{\vec{q}}_{2}\right)}{\left|\right|{\vec{q}}_{3}\left|\right|}.$$

The shortest vector ${\vec{r}}_{\mathrm{BPR}}$ connecting a point $\vec{p}$ to the line segment is then

(21) $${\vec{r}}_{\mathrm{BPR}}=\left\{\begin{array}{cc} {\vec{q}}_{2}-\vec{p}, & t\leq 0\\ {\vec{q}}_{2}+t{\vec{q}}_{3}-{\vec{p}}_{i}, & 0<t<1\\ {\vec{q}}_{3}-\vec{p}, & 1\leq t. \end{array}\right.$$

Hence, the sum of squares of two-norms

(22) $$\sum_{i}\left|\right|{\vec{r}}_{\mathrm{BPR},i}{\left|\right|}_{2}^{2}$$

for all ray-points correspondences $\left({\vec{v}}_{0,i},{\vec{p}}_{i}\right)$ must be minimized.

#### 3.5.4. Total Error

If the cameras and the projector are calibrated in the air, implying that we know the corresponding intrinsic parameters for each camera (projector) expressed in the same physical units, then all three vectors given by Equations (15) (17) and (21) are measured using the same physical units, and they may be joined together in an objective function for the optimization algorithm. Therefore, the total error, which defines the objective function to minimize, is

(23) $$\sum_{i}\left|\right|{\vec{r}}_{\mathrm{CPL},i}{\left|\right|}_{2}^{2}+\left|\right|{\vec{r}}_{\mathrm{FRS},i}{\left|\right|}_{2}^{2}+\left|\right|{\vec{r}}_{\mathrm{BPR},i}{\left|\right|}_{2}^{2}.$$

The value of (23) is minimized over the space of parameters, which includes the system axis ${\hat{a}}_{x}$, the pose parameters (a 3×3 rotation matrix S and a 3×1 translation vector u), as well as distances $d_{0}$ and $d_{1}$. For a nonlinear least-squares minimization scheme, we utilized the Levenberg–Marquardt optimization algorithm (i.e., Matlab’s lsqnonlin function [33]).

#### 3.5.5. Boundary Constraints

We proposed additional tweaks on the minimization of the objective function, i.e., we imposed boundary constraints on the optimization algorithm. A nonlinear least-squares fitting problem optionally may be bounded by upper and/or lower bounds. The components of an initial value $x_{0}$ that violate the bounds

(24) LB≤x≤UB,

where *x* is an output, LB is a lower bound, and UB is an upper bound, are reset to the interior of the box defined by the bounds. Components that satisfy the boundary constraint are not changed. We set boundaries for the distance $d_{0}$ between the camera’s (projector’s) extrinsic center and the glass interface, and, for thickness $d_{1}$ of the glass interface, the ranges of these two parameters are easily estimated (see Figure 1).

### 3.6. SL System Calibration

The purpose of geometric calibration is to determine all parameters that describe the image formation model of a particular SL system. Our proposed calibration method required two rounds of data acquisition: the first one was performed in-the-air without the shared protective glass, and the second one was performed underwater, with both using the same calibration object. The proposed geometric calibration of an underwater SL imaging system comprising an arbitrary number of cameras and projectors is performed as follows:

1. Calibrate all cameras and projectors in-the-air using a standard pinhole model with distortions as described in Section 3.4.
2. Acquire as many images of the calibration board in the water as is practical and process the data using the procedure of [21] to extract the calibration data.
3. Estimate the axis using Equation (14). In addition, for each position of the calibration board, estimate the initial pose of the calibration board w.r.t. the camera/projector frame using central approximation.
4. Use a numerical optimization with the objective function comprising the coplanarity error and of the frustum error (see Section 3.5) to estimate true relative poses and to refine the axis; this is performed separately for each position of the calibration board.
5. Use the numerical optimization with the complete objective function comprising the backprojection, the coplanarity, and the frustum errors to refine all parameters (see Section 3.5).

## 4. Evaluation

Evaluation of the proposed calibration method was achieved by performing several quantitative measurements. First, we computed the angular error of the estimated axis of the system; second, we computed the error in 3D (23); third, we computed the re-projection error; and, fourth, we computed the distance error for a plane fitted on the reconstructed calibration board. The errors are reported as a mean for each position of the calibration board w.r.t. the different glass interface. For the comparisons with the state-of-the-art techniques, we used the method proposed by Agrawal et al. [19] as the baseline.

### 4.1. Laboratory Setup

**Figure 6:**
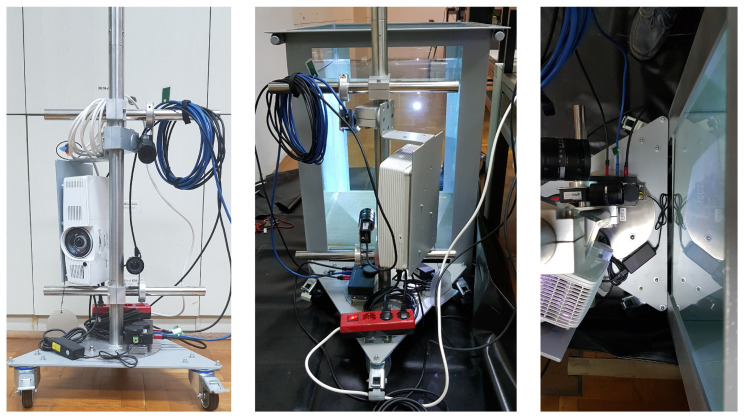
Laboratory setup. **Left**: SL scanner. **Middle**: side view. **Right**: top view.

The proposed method was evaluated on a laboratory setup comprising a water tank with a 300 L capacity filled with fresh water, a projector, and two cameras mounted on a fixed rig (see Figure 6). The water tank comprised four glass interfaces, with each interface having different properties. We labeled the interfaces with indices from I to IV: the interface I was the 8mm thick glass; the interface II was the 10mm thick glass; the interface III was the 10mm thick laminated glass; and the interface IV was the 12mm thick glass. The two cameras used were PointGrey’s Grasshopper 3 GS3-U3-23S6C-C cameras equipped with Fujinon HF12.5SA-1 lenses, and the projector used was an Acer S1383WHne.

### 4.2. Data Acquisition

**Figure 7:**
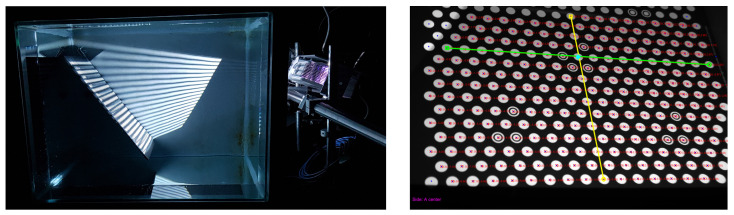
Underwater imaging (**left**: structured light projection; **right**: extraction of points’ coordinates).

Since an in-air calibration of both the camera and projector is a prerequisite, we first performed the geometrical calibration using the procedure of [21]. Then, we acquired the calibration data for four relative positions of the imaging system w.r.t. to the four glass interfaces; we performed this calibration once per each glass interface (see Section 4.1). For every position of the imaging system, the calibration board (Figure 3) was imaged underwater in at least five positions. The extraction of the calibration points (Figure 7, right) was performed using the procedure of [21], and the SL code (Figure 7, left) was MPS-coded using 20:21:25 ratios [32].

### 4.3. Axis Estimation

The axis of an axial system may be estimated from the proposed unified coplanarity constraint given by Equation (14) directly as $a_{1}$ or indirectly by the decomposition of the matrix E (see Section 3.3). We estimated the axis of the system directly from the coplanarity constraint. The mean angular error for the obtained axis of the system is presented in Table 1 for four different glass interfaces.

### 4.4. Errors in 2D and 3D

Three components form the total error in 3D: the coplanarity error, the backprojection error, and the frustum error (23). The total error in 3D is presented in Table 2 as a mean backprojection and coplanarity error for four different glass interfaces. Note that the frustum error was expected to be zero, since all calibration points needed to be within the field of view (FOV).

The re-projection error, i.e., the distance in pixels in the image frame between the observed 2D point and the re-projected 3D point, is computed using the AFP equation [19] for a two-layer flat refractive system. Note that the AFP equation was not used in the calibration procedure. Table 2 presents the mean and median values of the re-projection error for four different glass interfaces (tank sides I–IV).

To evaluate the 3D reconstruction quality, we also fit 3D planes onto the reconstructed 3D points for each position of the calibration board. Table 3 lists the mean absolute distances of the reconstructed 3D points for the ideal fitted plane.

## 5. Discussion

The calibrated imaging geometry is presented in Figure 5, where calibrated poses of the calibration board are shown. Backprojected rays were drawn for the position of the board w.r.t. the side I of the tank; rays in the air are displayed in red, green rays are the refracted rays in the glass interface, and blue rays are the refracted rays in the water.

**Figure 8:**
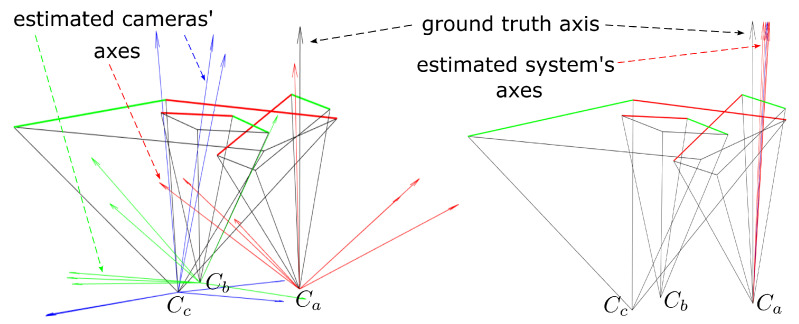
Estimated cameras’ axes for different views of the calibration board (before optimization); the estimates were used as an initial point in the optimization. **Left**: using the coplanarity constraint for a single camera/projector where red, green, and blue vectors correspond to axes acquired for each device in the system; **Right**: using the unified coplanarity constraint for a system where the axis of the system is denoted in red. The ground truth axis is denoted in black for both the left and the right case.

Regarding the axis estimation, Zoraja et al. [9] conjectured that the coplanarity constraint given by Equation (1) exhibits a coupling between the axis ${\hat{a}}_{x}$ and the pose parameters S and u. The coplanarity constraint of Equation (1) states that the points in 3D must lie on the POR, but, if we flatten the 3D space onto the POR, the constraint holds as well. Let $O=\mathrm{Null}{\left(a^{T}\right)}^{T}$ be a 3×2 matrix representing the orthographic projection of the 3D space onto the null space of ${\hat{a}}_{x}$. Then,

(25) $${\left(O({\left[a\right]}_{\times }v_{0})\right)}^{T}\left(O(Sp+u)\right)=v_{0}^{T}{\left[a\right]}_{\times }O^{T}O\left(Sp+u\right)=0$$

holds, thus indicating a possible instability in the solution, as points p are planar and losing one degree of freedom, and, due to coupling the null-space of Equation (4), are susceptible to noise. Zoraja et al. [9] analyzed the data provided by Agrawal et al. [19] by minimizing Equation (1) to start with many random initial axis directions, and they found at least three local minima having an average CPL error less than 1 mm. Axis estimations using the procedure of Agrawal et al. [19] are shown in Figure 8 on the left: in red are estimated axes of the camera a, in green are the estimated axes of the camera b, and in blue are the estimated axes of the projector c. The ground truth axis of the system is displayed in black. On the other hand, if the axis is estimated using the unified coplanarity constraint, (14) then the results are much more robust. The axis estimated in such way is a robust initial point for the proposed optimization algorithm, and this approach is presented in Figure 8 on the right: in red are the estimated axes of the system, and in black is the ground truth axis of the system.

Numerical values for mean angular errors (before optimization) w.r.t. different sides of the tank are listed in Table 1: the column Ours lists angles between the mean estimated axes of the system and the ground truth (the sub-column $\theta_{A}$ for axes was extracted from Equation (14), and the sub-column $\theta_{a}$ for axes was obtained by the decomposition of the essential matrix of the system), and the column Agrawal et al. [19] lists angles between the mean estimated axes of the single camera/projector obtained by the decomposition of the corresponding essential matrices [19]. The mean angular error of the estimated systems’ axes for each position of the SL system w.r.t. the tank’s side was 5.17deg (without considering the side III), which is acceptable and is a great improvement considering the error we obtained from Agrawal et al. [19] (see also Zoraja et al. [9]). Furthermore, system’s axis estimated by the unified coplanarity constraint was a good initial value for the optimization algorithm. Note that the angular error obtained w.r.t. the side III was greater than others due to the optical properties of the laminated glass, which we modeled as a single glass medium (without considering different optical properties for each layer of the laminated glass).

**Figure 9:**
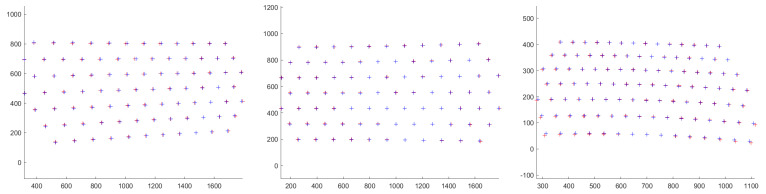
Extracted 2D points and corresponding re-projected 3D points for a single position of the calibration board in water (imaged over the 8mm thick glass interface): **left**—error for the camera a, **middle**—error for the camera b, and **right**—error for the projector. Red crosses represent re-projected 3D points in the camera (projector) frame, and blue crosses are the extracted 2D points.

The numerical results for the total error w.r.t. the different sides of the tank are listed in Table 2. Both the experimental backprojection and the coplanarity errors were less than one millimeter, which was sufficient for the 3D reconstruction using SL imaging when considering a working distance of up to 2 m. The numerical results for the re-projection error w.r.t. the different side of the tank are listed in Table 2; both the mean and median of the re-projection error are presented. For better visualization of the estimated re-projection error, we provide Figure 9 for a single position of the calibration board in water (imaged over the 8 mm thick glass interface). Notice that re-projection error was greater for the camera a, w.r.t. the camera b and the projector c, due to the greater angle between the optical axis and the axis of the system (see Figure 5). The re-projection error increased with the aforementioned angle.

The last quantitative measure is the distance error for a fitted plane. The numerical results for the proposed error w.r.t. the different sides of the tank are listed in Table 3. Considering the number of positions $N_{i}$ in which the calibration board was imaged, for every position (I–IV) of the imaging system, we reconstructed the calibration board and fitted the plane on the acquired dataset, thus acquiring the mean distance error. For positions where the angle δ between the axis ${\hat{a}}_{x}$ and the normal of the calibration board was minimal, the mean error was less than a millimeter. By increasing δ, the mean distance error increased.

Note that the error in 3D, the re-projection error, and the distance error for the fitted plane were greater for the pose where an imaging system observed the calibration board over the side III of the tank due to the optical properties of the laminated glass, which we modeled as a simple glass interface (disregarding reflections and refractions on multiple layers of the laminated glass).

## 6. Conclusions

We have presented a novel calibration procedure of a structured light underwater 3D imaging system using the theory of two-layer flat refractive geometry. The proposed calibration procedure is applicable to SL scanners with an arbitrary number of cameras and projectors, which share a common flat glass interface. It also uses a simple planar calibration board, which is readily available. The obtained experimental results demonstrated a low mean angular error for the estimation of the axis of the system using the proposed unified coplanarity constraint. This indicates that the proposed unified coplanarity constraint gives a robust initial estimate for the optimization algorithm. The experimentally assessed errors in 3D were less then one millimeter, which is sufficient for a 3D reconstruction and which indicates that the proposed calibration is applicable in the real world.

### Future Work

Given that the proposed system was evaluated in a laboratory with a tank simulating an underwater environment, our future work will focus on the design and implementation of a prototype of an underwater SL scanner that will be tested in a real underwater environment. We will also investigate the possibility of using a 3D calibration object comprising two planar boards at a $90^{{}^{\circ}}$ angle. Finally, we will investigate the robustness of the MPS-structured light pattern under conditions of increased turbidity.

## Figures and Tables

**Table 1:** Mean angular errors and standard deviation for the axis direction w.r.t. the true axis of the system (measured in air): column *i* lists sides of the tank; column $d_{1}$ lists corresponding glass thickness; column Ours lists error for the axis A estimated from the Equation (14) and for the axis a extracted by decomposition of the essential matrix $E_{1}$; column Agrawal et al. lists errors by utilizing the algorithm proposed by Agrawal et al. [19]. Sides II and III of the tank are both 10 mm thick; the exponent (a) denotes a real glass, while the exponent (b) denotes a laminated glass.

*i*	$d_{1}$	Ours		Agrawal et al. [19]		
	[mm]	θ${}_{A}$ [${}^{\circ }$]	θ${}_{a}\;$[${}^{\circ }$]	θ${}_{{\mathrm{CAM}}_{a}}\;$[${}^{\circ }$]	θ${}_{{\mathrm{CAM}}_{b}}\;$[${}^{\circ }$]	θ${}_{{\mathrm{PRJ}}_{c}}\;$[${}^{\circ }$]
I	8	4.85±0.73	4.83±0.71	81.22±18.36	78.44±17.04	47.00±38.30
II	10 ${}^{\left(a\right)}$	5.34±0.81	5.35±0.80	81.81±22.16	73.65±26.76	46.96±27.23
III	10 ${}^{\left(b\right)}$	17.38±16.72	17.11±16.58	79.16±32.19	79.04±17.59	84.76±6.22
IV	12	5.31±0.83	5.32±0.82	72.95±31.19	64.61±22.33	64.98±17.31

**Table 2:** Errors in 2D and 3D: column *i* lists sides of the tank, while column $d_{1}$ lists corresponding glass thickness; column Total Error (Mean) lists mean coplanarity and backprojection errors in 3D; column Re-Projection Error (Mean) lists mean errors in 2D for a corresponding camera (projector); and column Re-Projection Error (Median) lists median of errors in 2D. Sides II and III of the tank are both 10 mm thick; the exponent (a) denotes a real glass, while the exponent (b) denotes a laminated glass.

*i*	$d_{1}$	Total Error		Re-Projection Error			Re-Projection Error		
		(Mean) [mm] (23)		(Mean) [px]			(Median) [px]		
	[mm]	*e* ${}_{\mathrm{BPR}}$	*e* ${}_{\mathrm{CPL}}$	$e_{a}$	$e_{b}$	$e_{c}$	$e_{a}$	$e_{b}$	$e_{c}$
I	8	0.63	0.17	5.11	2.38	1.91	5.24	1.99	1.32
II	10 ${}^{\left(a\right)}$	0.47	0.13	4.22	2.01	1.80	4.47	1.72	1.23
III	10 ${}^{\left(b\right)}$	2.72	0.78	16.33	6.88	5.20	18.54	6.00	2.64
IV	12	0.50	0.14	4.12	2.19	1.66	4.16	1.97	1.24

**Table 3:** Fitting errors: column *i* lists sides of the tank; column $d_{1}$ lists corresponding glass thickness; column $N_{i}$ lists number of positions in which the calibration board was imaged; column Fitting Error (Mean) lists mean errors computed as distances from the fitted plane in 3D; and column Fitting Error (Median) lists median errors computed as distances from the fitted plane in 3D. Sides II and III of the tank are both 10 mm thick; the exponent (a) denotes a real glass, while the exponent (b) denotes a laminated glass.

*i*	$d_{1}$	$N_{i}$	Fitting Error (Mean) [mm]	Fitting Error (Median) [mm]
	[mm]		$\left[e_{j}\right]$, $j=1,\ldots ,N_{i}$	$\left[e_{j}\right]$, $j=1,\ldots ,N_{i}$
I	8	7	[1.83], [1.31], [0.60], [2.29], [1.08], [1.12], [1.69]	[1.34], [1.06], [0.46], [1.80], [0.90], [0.93], [1.39]
II	$10^{\;(a)}$	6	[1.18], [1.16], [0.84], [2.16], [1.43], [1.37]	[0.99], [0.95], [0.69], [1.68], [1.14], [1.07]
III	$10^{\;(b)}$	7	[2.70], [1.80], [2.42], [4.16], [1.76], [3.21], [3.68]	[2.36], [1.56], [2.13], [3.40], [1.55], [2.82], [3.07]
IV	12	6	[1.24], [1.09], [0.96], [2.12], [1.40], [1.30]	[1.04], [0.90], [0.80], [1.62], [1.12], [1.02]

## Data Availability

On request.

## Abbreviations

The following abbreviations are used in this manuscript:

ROV	Remotely Operated Vehicle
UAV	Underwater Autonomous Vehicle
SL	Structured Light
FRC	Flat Refraction Constraint
POR	Plane of Refraction
MPS	Multiple Phase Shift
SVD	Single Value Decomposition
AFP	Analytical Forward Projection
CPL	Coplanarity
BPR	Backprojection
FRS	Frustum
FOV	Field of View

## Appendix A

Here, we present the complete derivation of the coplanarity constraint individually for a single camera/projector and the derivation of the unified coplanarity constraint for an imaging system. The equation for the unified coplanarity constraint is derived w.r.t. the referent coordinate system, which we set to be the coordinate system of the camera a.

### Appendix A.1. Coplanarity Constraint for Camera (Projector) Using a Single Interface

A coplanarity constraint is given as:

(A1) $${\left({\left[a\right]}_{\times }v_{0}\right)}^{T}\left(Sp+u\right)=0.$$

Considering Equation (A1), the coplanarity constraint between the calibration object and the camera a may me derived as:

(A2) $$\begin{array}{cc} {\left({\left[a_{1}\right]}_{\times }v_{0_{a}}\right)}^{T}\left(S_{a}p_{a}+u_{a}\right) & =0\\ -v_{0_{a}}^{T}\left[{\left[a_{1}\right]}_{\times }\left(S_{a}p_{a}+u_{a}\right)\right] & =0\\ v_{0_{a}}^{T}\left[{\left[a_{1}\right]}_{\times }S_{a}p_{a}+{\left[a_{1}\right]}_{\times }u_{a}\right] & =0\\ v_{0_{a}}^{T}{\left[a_{1}\right]}_{\times }S_{a}p_{a}+v_{0_{1}}^{T}{\left[a_{1}\right]}_{\times }u_{a} & =0. \end{array}$$

By using the following identities

(A3) $$\begin{array}{cc} {\left[a_{1}\right]}_{\times }S_{a} & =E_{1} \end{array}$$

(A4) $$\begin{array}{cc} {}[a_{1}{]}_{\times }u_{a} & =h_{1} \end{array}$$

the Equation (A2) may be simplified as

(A5) $$v_{0_{a}}^{T}E_{1}p_{a}+v_{0_{a}}^{T}h_{1}=0.$$

The resulting linear system from Equation (A5) may be expressed in the matrix form:

(A6) $$\left[\begin{array}{cc} p_{a}{\left(1\right)}^{T}⊗v_{0_{a}}{\left(1\right)}^{T} & v_{0_{a}}{\left(1\right)}^{T}\\ p_{a}{\left(2\right)}^{T}⊗v_{0_{a}}{\left(2\right)}^{T} & v_{0_{a}}{\left(2\right)}^{T}\\ \vdots & \vdots \end{array}\right]\left[\begin{array}{c} E_{1}\left(:\right)\\ h_{1} \end{array}\right]\;=0,$$

where ⊗ denotes a Kronecker’s product, and $E_{1}\left(:\right)$ is the vector formed by stacking the columns of the essential matrix $E_{1}$.

Considering Equation (A1), the coplanarity constraint between the calibration object and the camera b is

(A7) $$\begin{array}{cc} {\left({\left[a_{2}\right]}_{\times }v_{0_{b}}\right)}^{T}\left(S_{b}p_{b}+u_{b}\right) & =0\\ -v_{0_{b}}^{T}\left[{\left[a_{2}\right]}_{\times }\left(S_{b}p_{b}+u_{b}\right)\right] & =0\\ v_{0_{b}}^{T}\left[{\left[R_{\mathrm{ab}}a_{2}\right]}_{\times }\left(R_{\mathrm{ab}}S_{a}p_{b}+R_{\mathrm{ab}}u_{a}+t_{\mathrm{ab}}\right)\right] & =0, \end{array}$$

and the coplanarity constraint between the calibration object and the projector c is

(A8) $$\begin{array}{cc} {\left({\left[a_{3}\right]}_{\times }v_{0_{c}}\right)}^{T}\left(S_{c}p_{c}+u_{c}\right) & =0\\ -v_{0_{c}}^{T}\left[{\left[a_{3}\right]}_{\times }\left(S_{c}p_{c}+u_{c}\right)\right] & =0\\ v_{0_{c}}^{T}\left[{\left[R_{\mathrm{ac}}a_{3}\right]}_{\times }\left(R_{\mathrm{ac}}S_{a}p_{c}+R_{\mathrm{ac}}u_{a}+t_{\mathrm{ac}}\right)\right] & =0. \end{array}$$

By using the following identities depicting rotations and translations between the coordinate systems a, b, and c,

$$\begin{array}{cccc} R_{\mathrm{ab}} & =R_{b}{R_{a}}^{T}\;\; & t_{\mathrm{ab}} & =-R_{b}C_{b}+R_{b}C_{a}\\ R_{\mathrm{ac}} & =R_{c}{R_{a}}^{T}\;\; & t_{\mathrm{ac}} & =-R_{c}C_{c}+R_{c}C_{a} \end{array}$$

and the identities for the rotations and translations between the aforementioned coordinate systems and the coordinate system of the calibration object (world frame),

$$\begin{array}{cccc} S_{b} & =R_{\mathrm{ab}}S_{a}\;\; & u_{b} & =R_{\mathrm{ab}}u_{a}+t_{\mathrm{ab}}\;\;\\ S_{c} & =R_{\mathrm{ac}}S_{a} & u_{c} & =R_{\mathrm{ac}}u_{a}+t_{\mathrm{ac}} \end{array}$$

the coplanarity constraint for the camera b w.r.t. the referent frame (A7) may be re-written as

(A9) $$\begin{array}{cc} v_{0_{b}}^{T}\left[{\left[R_{\mathrm{ab}}a_{1}\right]}_{\times }R_{\mathrm{ab}}S_{a}p_{b}+{\left[R_{\mathrm{ab}}a_{1}\right]}_{\times }R_{\mathrm{ab}}u_{a}+{\left[R_{\mathrm{ab}}a_{1}\right]}_{\times }t_{\mathrm{ab}}\right] & =0\\ v_{0_{b}}^{T}\left[R_{\mathrm{ab}}\left({\left[a_{1}\right]}_{\times }S_{a}p_{b}\right)+R_{\mathrm{ab}}\left({\left[a_{1}\right]}_{\times }u_{a}\right)+{\left[R_{\mathrm{ab}}a_{1}\right]}_{\times }t_{\mathrm{ab}}\right] & =0\\ v_{0_{b}}^{T}R_{\mathrm{ab}}\left({\left[a_{1}\right]}_{\times }S_{a}p_{b}\right)+v_{0_{b}}^{T}R_{\mathrm{ab}}\left({\left[a_{1}\right]}_{\times }u_{a}\right)+v_{0_{b}}^{T}{\left[R_{\mathrm{ab}}a_{1}\right]}_{\times }t_{\mathrm{ab}} & =0, \end{array}$$

and the coplanarity constraint for the projector c w.r.t. the referent frame (A8) may be re-written as

(A10) $$\begin{array}{cc} v_{0_{c}}^{T}\left[{\left[R_{\mathrm{ac}}a_{1}\right]}_{\times }R_{\mathrm{ac}}S_{a}p_{c}+{\left[R_{\mathrm{ac}}a_{1}\right]}_{\times }R_{\mathrm{ac}}u_{a}+{\left[R_{\mathrm{ac}}a_{1}\right]}_{\times }t_{\mathrm{ac}}\right] & =0\\ v_{0_{c}}^{T}\left[R_{\mathrm{ac}}\left({\left[a_{1}\right]}_{\times }S_{a}p_{c}\right)+R_{\mathrm{ac}}\left({\left[a_{1}\right]}_{\times }u_{a}\right)+{\left[R_{\mathrm{ac}}a_{1}\right]}_{\times }t_{\mathrm{ac}}\right] & =0\\ v_{0_{c}}^{T}R_{\mathrm{ac}}\left({\left[a_{1}\right]}_{\times }S_{a}p_{c}\right)+v_{0_{c}}^{T}R_{\mathrm{ac}}\left({\left[a_{1}\right]}_{\times }u_{a}\right)+v_{0_{c}}^{T}{\left[R_{\mathrm{ac}}a_{1}\right]}_{\times }t_{\mathrm{ac}} & =0. \end{array}$$

By using the identities from Equations (A3) and (A4), we may simplify Equations (A9) and (A10) as

(A11) $$v_{0_{b}}^{T}R_{\mathrm{ab}}E_{1}p_{b}+v_{0_{b}}^{T}R_{\mathrm{ab}}h_{1}+v_{0_{b}}^{T}{\left[R_{\mathrm{ab}}a_{1}\right]}_{\times }t_{\mathrm{ab}}=0$$

and

(A12) $$v_{0_{c}}^{T}R_{\mathrm{ac}}E_{1}p_{c}+v_{0_{c}}^{T}R_{\mathrm{ac}}h_{1}+v_{0_{c}}^{T}{\left[R_{\mathrm{ac}}a_{1}\right]}_{\times }t_{\mathrm{ac}}=0,$$

where Equation (A11) denotes the coplanarity constraint between the calibration object and the camera b w.r.t. the referent frame, and Equation (A12) denotes the coplanarity constraint between the calibration object and the projector c w.r.t. the referent frame.

### Appendix A.2. Unified Coplanarity Constraint for System Using a Single Interface

Equations (A5), (A11), and (A12) denote the coplanarity constraints of camera a, camera b, and projector c w.r.t. the referent frame, respectively. The third terms of Equations (A11) and (A12) may be reformed, which, for Equation (A11), results in

$$\begin{array}{cc} v_{0_{b}}^{T}{\left[R_{\mathrm{ab}}a_{1}\right]}_{\times }t_{\mathrm{ab}} & =v_{0_{b}}^{T}{\left[R_{\mathrm{ab}}a_{1}\right]}_{\times }\left(R_{\mathrm{ab}}{R_{\mathrm{ab}}}^{T}t_{\mathrm{ab}}\right)\\ & =v_{0_{b}}^{T}R_{\mathrm{ab}}\left[{\left[a_{1}\right]}_{\times }\left({R_{\mathrm{ab}}}^{T}t_{\mathrm{ab}}\right)\right]\\ \; & =a_{1}^{T}\left[\left({R_{\mathrm{ab}}}^{T}t_{\mathrm{ab}}\right)\times {\left(v_{0_{b}}^{T}R_{\mathrm{ab}}\right)}^{T}\right]\\ \; & =a_{1}^{T}\left[\left({R_{\mathrm{ab}}}^{T}t_{\mathrm{ab}}\right)\times \left({R_{\mathrm{ab}}}^{T}v_{0_{b}}\right)\right]\\ \; & =a_{1}^{T}\left[{R_{\mathrm{ab}}}^{T}\left(t_{\mathrm{ab}}\times v_{0_{b}}\right)\right]\\ \; & ={\left[{R_{\mathrm{ab}}}^{T}\left(t_{\mathrm{ab}}\times v_{0_{b}}\right)\right]}^{T}a_{1.} \end{array}$$

Similarly, the third term of Equation (A12) may be reformed as

$$\begin{array}{cc} v_{0_{c}}^{T}{\left[R_{\mathrm{ac}}a_{1}\right]}_{\times }t_{\mathrm{ac}} & =v_{0_{c}}^{T}{\left[R_{\mathrm{ac}}a_{1}\right]}_{\times }\left(R_{\mathrm{ac}}{R_{\mathrm{ac}}}^{T}t_{\mathrm{ac}}\right)=\cdots ={\left[{R_{\mathrm{ac}}}^{T}\left(t_{\mathrm{ac}}\times v_{0_{c}}\right)\right]}^{T}a_{1.} \end{array}$$

Considering the reformation of the third term in both Equations (A11) and (A12), both linear systems may be re-written as

(A13) $$\begin{array}{cc} v_{0_{b}}^{T}R_{\mathrm{ab}}E_{1}p_{b}+v_{0_{b}}^{T}R_{\mathrm{ab}}s_{1}+{\left[{R_{\mathrm{ab}}}^{T}\left(t_{\mathrm{ab}}\times v_{0_{b}}\right)\right]}^{T}a_{1} & =0 \end{array}$$

(A14) $$\begin{array}{cc} v_{0_{c}}^{T}R_{\mathrm{ac}}E_{1}p_{c}+v_{0_{c}}^{T}R_{\mathrm{ac}}s_{1}+{\left[{R_{\mathrm{ac}}}^{T}\left(t_{\mathrm{ac}}\times v_{0_{c}}\right)\right]}^{T}a_{1} & =0. \end{array}$$

The matrix form of Equation (A11) is

(A15) $$\left[\begin{array}{ccc} p_{b}{\left(1\right)}^{T}⊗\left(v_{0_{b}}{\left(1\right)}^{T}R_{\mathrm{ab}}\right) & v_{0_{b}}{\left(1\right)}^{T}R_{\mathrm{ab}} & {\left[{R_{\mathrm{ab}}}^{T}\left(t_{\mathrm{ab}}\times v_{0_{b}}\left(1\right)\right)\right]}^{T}\\ p_{b}{\left(2\right)}^{T}⊗\left(v_{0_{b}}{\left(2\right)}^{T}R_{\mathrm{ab}}\right)\; & v_{0_{b}}{\left(2\right)}^{T}R_{\mathrm{ab}} & {\left[{R_{\mathrm{ab}}}^{T}\left(t_{\mathrm{ab}}\times v_{0_{b}}\left(2\right)\right)\right]}^{T}\\ \vdots & \vdots & \vdots \end{array}\right]\left[\begin{array}{c} E_{1}\left(:\right)\\ s_{1}\\ a_{1} \end{array}\right]=0,$$

and the matrix form of Equation (A12) is

(A16) $$\left[\begin{array}{ccc} p_{c}{\left(1\right)}^{T}⊗\left(v_{0_{c}}{\left(1\right)}^{T}R_{\mathrm{ac}}\right) & v_{0_{c}}{\left(1\right)}^{T}R_{\mathrm{ac}} & {\left[{R_{\mathrm{ac}}}^{T}\left(t_{\mathrm{ac}}\times v_{0_{c}}\left(1\right)\right)\right]}^{T}\\ p_{c}{\left(2\right)}^{T}⊗\left(v_{0_{c}}{\left(2\right)}^{T}R_{\mathrm{ac}}\right)\; & v_{0_{c}}{\left(2\right)}^{T}R_{\mathrm{ac}} & {\left[{R_{\mathrm{ac}}}^{T}\left(t_{\mathrm{ac}}\times v_{0_{c}}\left(2\right)\right)\right]}^{T}\\ \vdots & \vdots & \vdots \end{array}\right]\left[\begin{array}{c} E_{1}\left(:\right)\\ s_{1}\\ a_{1} \end{array}\right]=0.$$

Considering linear systems, posed by Equations (A6), (A15), and (A16), by denoting the coplanarity constraints between the calibration object and the corresponding camera/projector frame w.r.t. the referent frame, we obtain the unified coplanarity constraint of Equation (14) for the imaging system w.r.t. the referent frame.
